# Strategic single point mutation yields a solvent- and salt-stable transaminase from *Virgibacillus* sp. in soluble form

**DOI:** 10.1038/s41598-018-34434-3

**Published:** 2018-11-06

**Authors:** Benedetta Guidi, Matteo Planchestainer, Martina Letizia Contente, Tommaso Laurenzi, Ivano Eberini, Louise J. Gourlay, Diego Romano, Francesca Paradisi, Francesco Molinari

**Affiliations:** 1 Department of Food, Environmental and Nutritional Sciences (DeFENS), University of Milan, Milan, Italy; 2 School of Chemistry, University of Nottingham, University Park, Nottingham, NG& 2RD UK; 3 Department of Biosciences, University of Milan, Milan, Italy; 4 Department of Pharmacological and Biomolecular Sciences (DiSFeB), University of Milan, Milan, Italy

## Abstract

A new transaminase (*Vb*TA) was identified from the genome of the halotolerant marine bacterium *Virgibacillus* 21D. Following heterologous expression in *Escherichia coli*, it was located entirely in the insoluble fraction. After a single mutation, identified via sequence homology analyses, the *Vb*TA T16F mutant was successfully expressed in soluble form and characterised. *Vb*TA T16F showed high stability towards polar organic solvents and salt exposure, accepting mainly hydrophobic aromatic amine and carbonyl substrates. The 2.0 Å resolution crystal structure of *Vb*TA T16F is here reported, and together with computational calculations, revealed that this mutation is crucial for correct dimerisation and thus correct folding, leading to soluble protein expression.

## Introduction

Transaminases (TAs, also known as aminotransferases) have been extensively studied in recent years for their potential as biocatalysts^1–3^. TAs require pyridoxal phosphate (PLP) as a coenzyme and catalyse the transfer of an amino group from an amino donor to a carbonyl acceptor, thus generating new amines and carbonyls, often with high stereoselectivity. Major efforts have been devoted to the discovery of new TAs and the evolution of known proteins for obtaining biocatalysts with selectivity and robustness fit for preparative biotransformations. To date, more than 60 microbial TAs have been described as potential biocatalysts, of which about 20 have been identified only in the last two years^4^. A number of examples of the evolution by protein engineering of natural TAs into more stable and more selective enzymes, with larger substrate scopes have also been reported^5–10^. This endeavor was made possible by the availability of new TA crystal structures that have provided a better insight into the functional mechanisms of these enzymes^1,11^. In addition, immobilisation of TAs has been shown to enable catalysis under practical conditions (in the presence of co-solvents, stoichiometric amounts of the amine donor) in continuous reactors, where the reaction can be performed with better productivity and long-term stability of the biocatalyst^12–14^.

Extremophile microorganisms (microbial species withstanding extreme conditions, such as low/high temperatures, low/high pH, high ionic strength and high hydrostatic pressure) have great potential as a source of enzymes that are active under harsh conditions^15^. In particular, marine microorganisms are known to produce enzymes characterised by outstanding tolerance to organic solvents and high salt concentrations^16^. The recruitment of new enzymes from marine microorganisms as selective and robust biocatalysts is highly attractive. A TA from the halo-adapted bacterium *Halomonas elongata* proved tolerant to the presence of co-solvents up to 20% and showed remarkable stability under operating conditions^17^. In this context, different biocatalytic activities (ketoreductase, esterase, and transaminase) have been reported in marine bacteria, such as *Virgibacillus* sp. 21D, a strain that grows in NaCl concentrations of up to 12%^18^.

In this current study, a TA gene (*Vb*TA) identified from the genome of *Virgibacillus* sp. 21D was codon optimised and expressed in *E. coli*, however, the protein was entirely found in inclusion bodies under all conditions studied. To address this problem, we successfully adopted a protein engineering approach, based on the mutation of a single residue (T16F) that resulted in sufficiently soluble expression of recombinant *Vb*TA T16F in *Escherichia coli*. Here, we report the functional and structural characterisation of the mutant enzyme. Analysis of the *Vb*TA T16F crystal structure, together with molecular modelling, provides an insight into the role of F16 in stabilising the tertiary and quaternary structure of *Vb*TA T16F.

## Results and Discussion

### Production of soluble VbTA after protein engineering

Protein sequences of TAs from *Vibrio fluvialis* (*Vf*TA), *Chromobacterium violaceum* (*Cv*TA), and *Halomonas elongata* (*He*TA) were used to search for homologous genes in the genome of *Virgibacillus* sp. 21D; this analysis detected a sequence (*Vb*TA) with an identity of 38% and 55% similarity with the *He*TA sequence, and 36% similarity with *Vf*TA and *Cv*TA genes.

**Figure 1:**
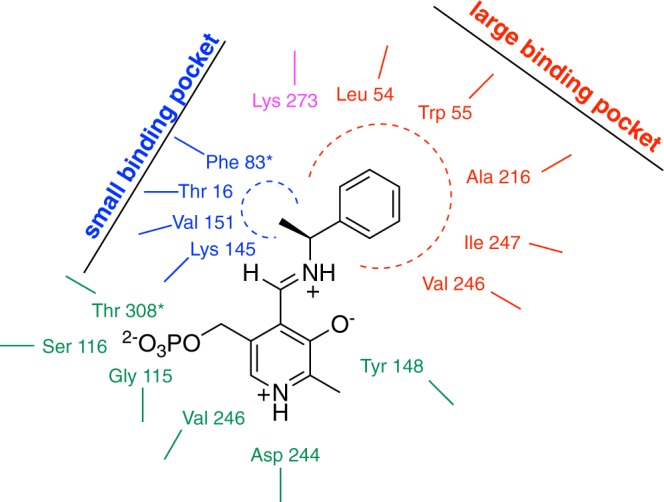
Schematic representation of the small and large binding pockets of *VbT*A. Phe 83* and Thr 308* belongs to the second subunit. Asp 244, Val 246 and Tyr 148 (in green) stabilise PLP aromatic ring; Gly 115, Ser 116, and Thr 308* coordinate the PLP phosphate group; whereas Lys 273 is the catalytic residue. The model is based on the alignment analysis and the molecular modeling based on the resolved structure of the amino transferase from *V. fluvialis* (PDB: 4E3Q)^37^.

The selected 1350 bp gene encodes for a protein of 448 amino acid residues. Classification of PLP-dependent enzymes is primarily based on fold type^19^; e.g., fold type I, also referred to as the ‘aspartate aminotransferase superfamily’, which combines the highest quantity and diversity of members^20^. Transaminases are additionally divided into six classes depending on common structural features and sequence similarity; *Vb*TA, according to its sequence, belongs to fold type I and the class III subgroup. Based on sequence alignments, it was possible to identify three highly conserved residues (see ConSurf analysis, Supplementary Figure 1) that bind PLP: D24 and Y148, involved in salt bridge and hydrogen bond formation with PLP, respectively, and K273 that forms a Schiff base with PLP (Fig. 1).

The codon optimised *Vb*TA gene sequence was cloned into different conventional *E. coli* expression systems (see Materials for details) and expressed under different fermentation settings (temperature, expression time, and inducer concentration) using a Mutisimplex optimization design^21^, resulting in the accumulation of expressed protein as insoluble inclusion bodies, under all conditions tested. The halophilic archaeon host *Haloferax volcanii*^22^ was also tested to optimise the marine transaminase expression, without any significant improvement.

While insoluble expression can be due to incompatibility of the host cytoplasmic environment, it is also known that misfolding can be linked to specific mutations in the sequence^23^. All fold type-I TAs reported in literature exist as dimers or tetramers and are generally produced as soluble proteins^1^. Therefore, the *VbT*A sequence was further analysed to gain insight into its structure and lack of solubility. Previous structural studies, whereby the active site of a *He*TA homology model was probed by site directed mutagenesis to investigate the enzyme affinity for specific aromatic substrates^24^, highlighted that F18 (corresponding to T16 in *VbT*A) is a key active site residue in the catalytic pocket. It forms a strong interaction with a second phenylalanine (F84, contributed by the second monomer), thus stabilizing the compact dimeric structure. *He*-TA F18A was generated at the time to increase the size of the small pocked to shift the activity of the enzyme towards bulky-bulky substrates, however this variant was found to be completely insoluble (data not shown), suggesting that the aromatic side chain at position 16 was essential for the correct folding of the protein. Furthermore, using the program ConSurf (http://consurf.tau.ac.il)^25^, sequence alignments made with around 500 homologous protein sequences showed that this position is highly conserved for aromatic residues (76.8% Phe, 16.3% Tyr, see Supplementary Figures 1 and 2). Therefore, based on the hypothesis that dimerisation is critical for solubility, and on the fact that amino acids involved in the structural stabilisation of the functional homodimer must be evolutionary preserved, to some extent, the mutant *Vb*TA T16F was produced.

Expression of the N-terminal His-tagged transaminase was performed in a conventional BL21 DE3 *E. coli* strain, transformed with the expression vector pET100-D-TOPO and its expression under different fermentation conditions was compared by SDS-PAGE (Supplementary Figure 3). The best results were achieved in ZYM-5052 auto-induction medium, with incubation at 30 °C, with shaking at 150 rpm, for 24 h. *Vb*TA T16F migrated with a MW of 50–55 kDa in agreement with its calculated MW of 53.2 kDa (including 4.1 kDa corresponding to the His-tag). In order to determine the quaternary arrangement of *Vb*TA T16F, size-exclusion chromatography experiments were carried out, resulting in an estimated MW of 104 kDa, corresponding to a dimeric quaternary structure (theoretical value 106 kDa).

### Characterization of transaminase activity

A standard assay, using (*S*)-1-phenylethylamine as the amino donor and pyruvate as the amino acceptor, was used to determine the transaminase activity of *Vb*TA T16F^26^. The highest activity was observed at pH 8.0 and 45 °C, with 60% of the initial activity still maintained at 60 °C. As described for the first time by Ikai *et at*.^27^, this mesophilic profile could be related to the high aliphatic index (94.44) of *Vb*TA T16F. Stability tests at different pHs and temperatures showed that the enzyme is highly stable at temperatures up to 45 °C when stored at pH 8.0 (see Supplementary Figure 4 for details).

**Figure 2:**
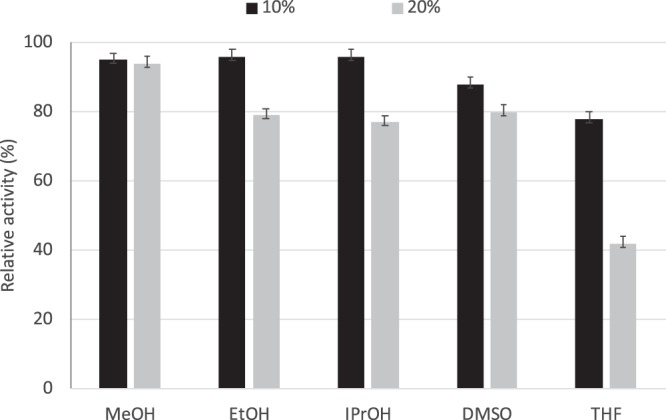
Stability of *Vb*TA T16F after storage for 2 days at 25 °C, in the presence of different solvents. Data are reported as relative percentage activity, in comparison with the control in standard conditions. Results are reported as the average of triplicate measurements.

*Virgibacillus* sp. strain 21D was isolated from a deep hypersaline anoxic basin characterised by the presence of high concentration of MgCl_2_ (5 M)^18^. This strain also grows in presence of NaCl concentrations up to 1.5 M, which may imply halotolerant behavior of its enzymes. As previously observed, enzymes from halotolerant microorganisms are stable in conditions of high ionic strength and co-solvents, which are often crucial for the reaction or storage medium in biocatalysis. Stability towards organic solvents (Fig. 2) was relatively high in most solvents tested; the enzyme, stored at 25 °C for 2 days in the presence of 20% *(v/v)* methanol (MeOH) maintained 94–95% of its initial activity. Activity of *Vb*TA T16F in the presence of 10% *(v/v)* MeOH was nearly unaffected and around 50% activity was observed in the presence of 20% *(v/v)* DMSO. This tolerance towards polar organic solvents, commonly employed for solubilising hydrophobic substrates, is quite remarkable and significantly better than previously reported for other TAs^1^.

**Figure 3:**
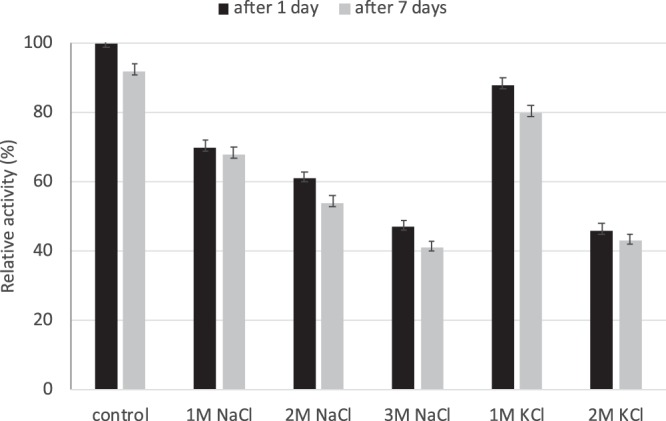
Stability of *VbTA* T16F at different salt concentrations. Data are reported as relative percentage activity in comparison with the control in standard conditions. Results are reported as the average of triplicate measurements.

Stability of *Vb*TA T16F in the presence of different concentrations of NaCl and KCl is reported in Fig. 3. *VbTA* T16F was stable and exhibited 63–65% of its initial activity after 7 days in the presence of 1 M NaCl (5.84%) and up to 80% in the presence of 1 M KCl (7.45%); even at 3 M NaCl (17.5%) still 40% of residual activity was retained after 1 week.

Under optimal reaction conditions, the maximum turnover number (*k*_cat_) of *Vb*TA T16F was 0.099 s^−1^ and measured Michaelis–Menten constants (K_M_) were 1.9 mM and 10.7 mM for (*S*)-1-phenylethylamine and pyruvate, respectively (see Supporting Information for further details).

### Substrate scope

Different amino acceptors and donors were evaluated under standard conditions (Table 1) to evaluate the substrate scope of *Vb*TA T16F.

**Table 1:** Substrate scope of *VbTA* T16F.

Amino donor^a^	Conversion (%)	Amino acceptor^b^	Conversion (%)
	43		<5
	71		
	n.d.		
	47		49
	95		94
	70		65
	90		78
	75		81
	86		90
	74		93
	90		91
	n.d.		n.d.
	86		88
	n.d.		
	n.d.		n.d.
	n.d.		n.d.
	n.d.		

Each reaction was performed in triplicate and the results are reported as the average of the data obtained after 24 h. Substrates were prepared in methanol solution to guarantee the correct concentration of the substrate in the reaction mixture (final concentration 10% *(v/v)* MeOH). Final conversions were determined by HPLC. ^a^Amino donor concentration was kept constant at 10 mM, and 10 mM pyruvate was used as the amino acceptor; ^b^amino acceptor concentration was kept constant at 10 mM; L-alanine was used as the amino donor. n.d. = non-detectable in the tested condition.

Hydrophobic substrates bearing aromatic groups were the preferred substrates, both as amino donors and acceptors, whereas small aliphatic substrates and keto-sugars were not generally converted; pyruvate/alanine were among the only small polar substrates accepted. *Vb*TA T16F showed a complete *S*-enantioselectivity toward 1-phenylethylamine.

### The crystal structure of VbTA T16F

In order to investigate the structural bases that govern the improved solubility of the heterologously expressed T16F mutant, the 3D structure of *Vb*TA T16F was solved at 2 Å resolution by X-ray crystallography. *Vb*TA T16F crystallised with one monomer in the asymmetric unit (Matthews coefficient (V_M_) of 2.68 A^3^/Da, with an estimated solvent content of 54.1%. Electron density was evident for residues 2 to 444 with no gaps. One molecule of PLP was bound at the active site, although it does not form a covalent aldimine bond with the catalytic lysine residue (K273), as seen in the crystal structures of many other PLP-dependent enzymes (See Supplementary Figure 5).

**Figure 4:**
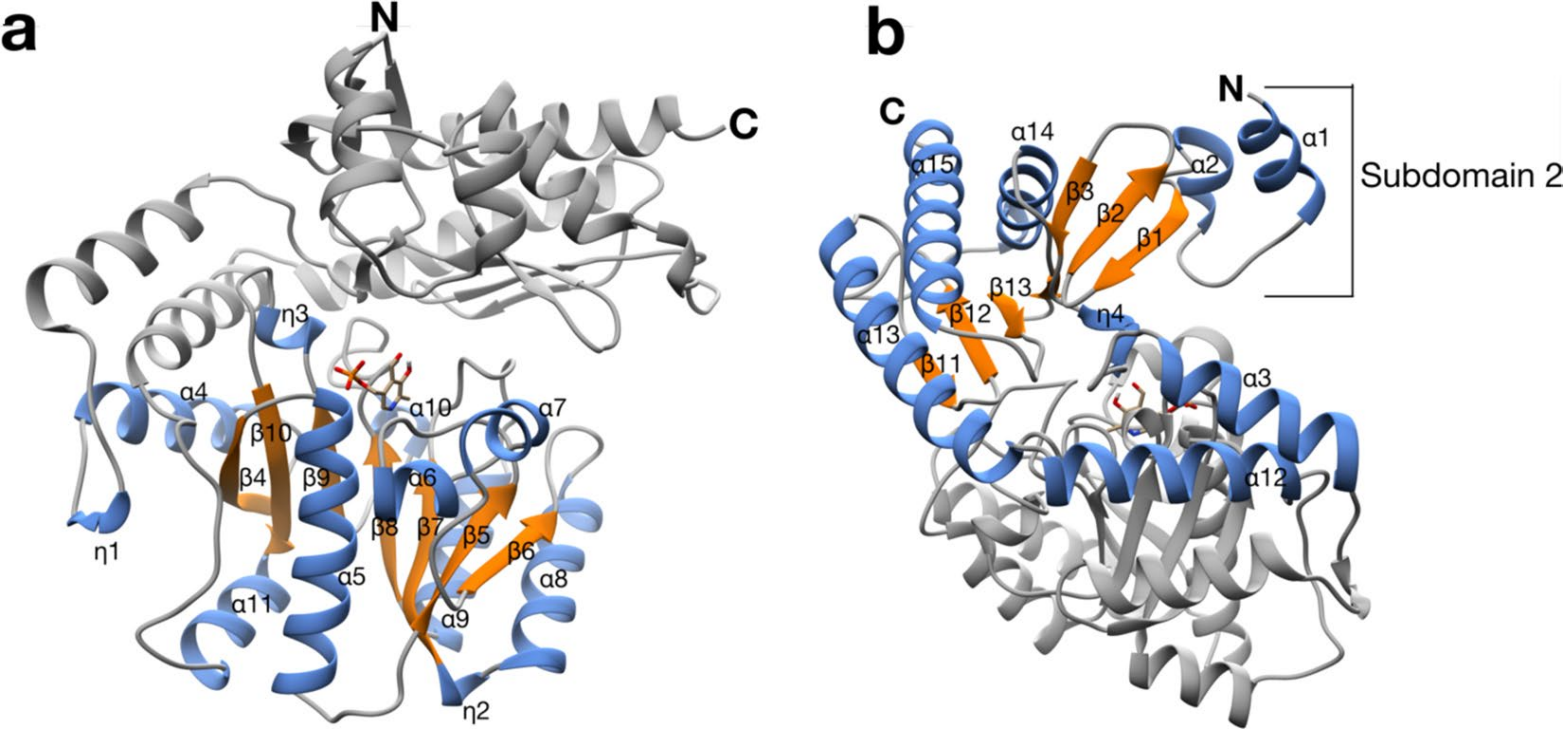
The crystal structure of the *Vb*TA T16F monomer. Two alternative views of the *Vb*TA T16F monomer, illustrating the secondary structure organisation of the two domains (cartoon representation). (**a**) the secondary structure elements of domain 1 are highlighted in orange (α-helices) and blue (β-strands). PLP bound to the active site is indicated as sticks. For clarity, domain 1 is shaded in grey; (**b**) the secondary structure elements of domain 2 are colored. For clarity, domain 1 is shaded in grey. This figure was generated using Chimera^32^.

The *Vb*TA T16F monomer possesses the canonical class III *(S)*-selective ω-aminotransferase fold, comprising two domains: a PLP-dependent transferase-like domain (residues 81 to 313) that hosts a central, seven-stranded mixed β-sheet (β4-β10-β9-β8-β7-β5-β6), with β-strand 10 being antiparallel to the rest, surrounded by 8 α-helices and three 3^10^ helices (η1-3) (Fig. 4a); ii) domain 2 (residues 2-80; 314–444) that contains a small N-terminal sub-domain (residues 2 to 49; α1-α2-β1-β2-β3) (Fig. 4b).

**Figure 5:**
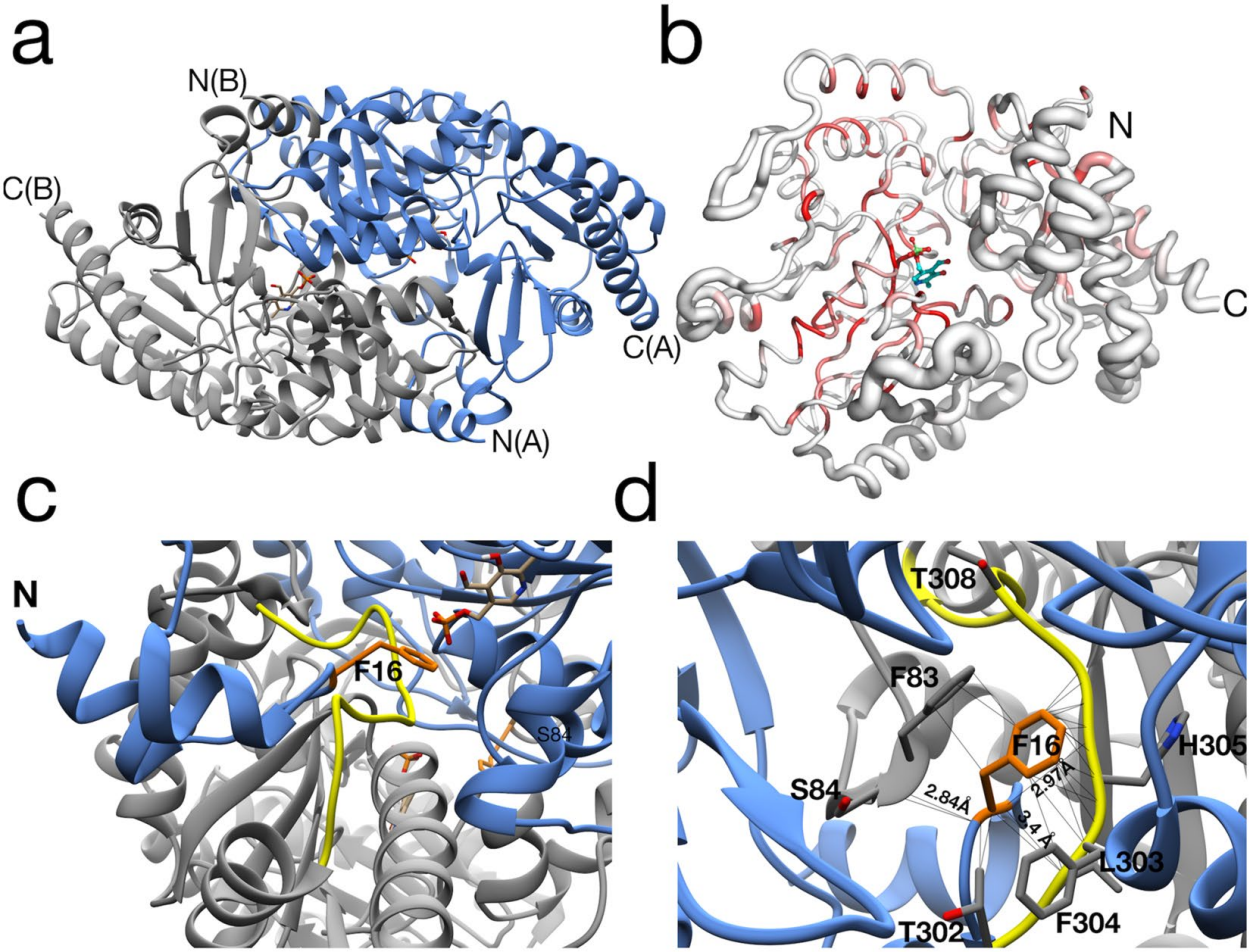
The *Vb*TA T16F dimer. (**a**) Quaternary organisation of *Vb*TA T16F, showing chains A (blue) and B (grey). The N- and C-termini are indicated and PLP is shown in sticks; (**b**) Secondary structure of *Vb*TA T16F shown in sausage representation, as automatically generated by ENDscript 2.0 (http://endscript.ibcp.fr)^29^. Structure conservation with 125 structure homologs deposited in the PDB is indicated by ribbon thickness, with regions of low conservation being thicker than highly conserved regions (thin regions). Sequence identity is indicated by red coloring; the redder the residue, the more conserved it is. The N- and C-termini are indicated and PLP is shown in sticks; (**c**) the F16 mutation (orange sticks) and bound PLP (sticks, atom colouring) in chain A are highlighted. The active site loop region (residues 298–313) in chain B, located between α11 and α12 is shown in yellow, and **d)** Detailed view of the interaction network (<4 Å; grey lines) between F16 (orange sticks) and residues F83, S84 and 302–305 (grey sticks) of the opposing monomer. The active site loop region (residues 298–313) in chain B is coloured yellow. Bond distances (Å) for the three hydrogen bonds are indicated. Panels A, C and D were generated using Chimera^32^, whereas panel B was generated using MacPymol 2.0.6.

In agreement with the majority of class III TAs, *Vb*TA T16F forms a dimer, generated between *Vb*TA T16F chain A and its symmetry-related monomer (180° rotation) (Fig. 5a). Dimerisation covers an interaction surface area of 5463 A^2^, involving 103 residues from each monomer and 48 hydrogen bonds (as calculated using PDBsum)^28^.

The closest structural homolog to *Vb*TA T16F is the apo-form of the omega transaminase from *Chromobacterium violaceum* (PDB entry 4A6R), with a sequence identity of 37.5% over 432/443 aligned residues (RMSD value = 1.3 Å)^11^. The sequence and structural conservation of *Vb*TA T16F with all homologous structures deposited in the PDB was assessed using ENDscript 2.0 (http://endscript.ibcp.fr)^29^. 125 deposited unique structures (sequence conservation > 30%) were superimposed with *Vb*TA T16F. As expected, the highest conservation was located to the PLP-binding domain residues; the least conservation was observed in flexible loop regions, and in particular in the N-terminal domain that mediates dimerization (Fig. 5b).

Based on the availability of a holo-, two apo-forms and intermediate states of *Cv*TA, Humble *et al*., demonstrated for the first time (for a transaminase) that, upon PLP binding, the enzyme undergoes extensive structural rearrangements in three loop regions that form the architecture of the active site^11^. In particular, the N-terminal subdomain of domain 2 completely refolds, as indicated by the lack of electron density in the apo-form of the *Cv*TA crystal structure (PDB entry 4A6R). Correct structural reorganisation of these loops was shown to be essential to form the catalytic pocket at the dimer interface.

F16 is located in a critical position, at the center of the 14-residue loop, connecting α1 to α2 of subdomain 2 (Fig. 5c,d). This loop clasps between α11 (domain 1) and α12 (domain 2) of the opposing monomer, locking the dimer together (Fig. 5c). F16 has a clear role in stabilising the dimer interface, making 26 contacts (<4 Å) with main- and side- chain atoms from six residues in the opposing monomer, including three hydrogen bonds with the carbonyl oxygen atoms of L303 (3.4 Å) and H305 (2.97 Å), and the nitrogen atom of the α-amine group of S84 (2.84 Å) (Fig. 5d). Such interactions result in the stabilisation of a second loop (residues 298–313) that contributes to the active site. The correct positioning of this loop is essential as it houses the highly conserved residue T308 that binds PLP *via* two hydrogen bonds between the phosphate oxygen atom (O2P) of PLP and the main chain amide nitrogen atom (length 2.78 Å) and side chain hydroxyl atom (length 2.64 Å) of T308. Furthermore, T308, in turn, also hydrogen bonds (length 2.68 Å) to the catalytic lysine residue K273 (NZ atom) *via* its side chain hydroxyl atom (See Supplementary Figure 5). These observations indicate that the loss of the F16 interaction network by substitution with threonine would result in significant destabilisation of the dimer interface, as confirmed in structural bioinformatics calculations described below.

### Stability and affinity calculations (wt versus T16F)

To evaluate the stability of monomeric and homodimeric forms of *Vb*TA, we performed *in silico* mutagenesis simulations and evaluated protein stability and affinity (Table 2).

**Table 2:** *In silico* mutagenesis and affinity/stability analysis and calculations.

T16F	Δaffinity (kcal/mol)	Δstability (kcal/mol)
Homodimer	−14.23	−19.88
Monomer	—	−4.82

Δaffinity values report the change in binding affinity within protomers between the wild type and *Vb*TA T16F mutant; Δstability values report the changes in protein (monomer or homodimer) stability occurring after the mutation; stability is defined as the difference in energy between the folded and unfolded states. Δaffinity and Δstability data are calculated using an implicit solvent MM-GBSA method.

The introduction of the T16F mutation significantly increases the protein stability, with a contribution of 4.82 kcal/mol computed for the monomeric forms. Since the F16T mutation is located at the dimerisation interface of *Vb*TA, its impact is significant for the homodimerisation process. The affinity of the protomers for one another differs by 14.23 kcal/mol between the wild type *Vb*TA and the F16T mutant, suggesting that the latter has a propensity to dimerise.

The stability of the *Vb*TA T16F dimer is 10.24 kcal/mol higher than mutant monomer (2 protomers × 4.82 kcal/mol), suggesting that dimerisation can increase T16F mutant stabilisation not only through intramolecular but also via intermolecular interactions (5.12 kcal/mol protomer).

### *In silico* solvent analysis

**Figure 6:**
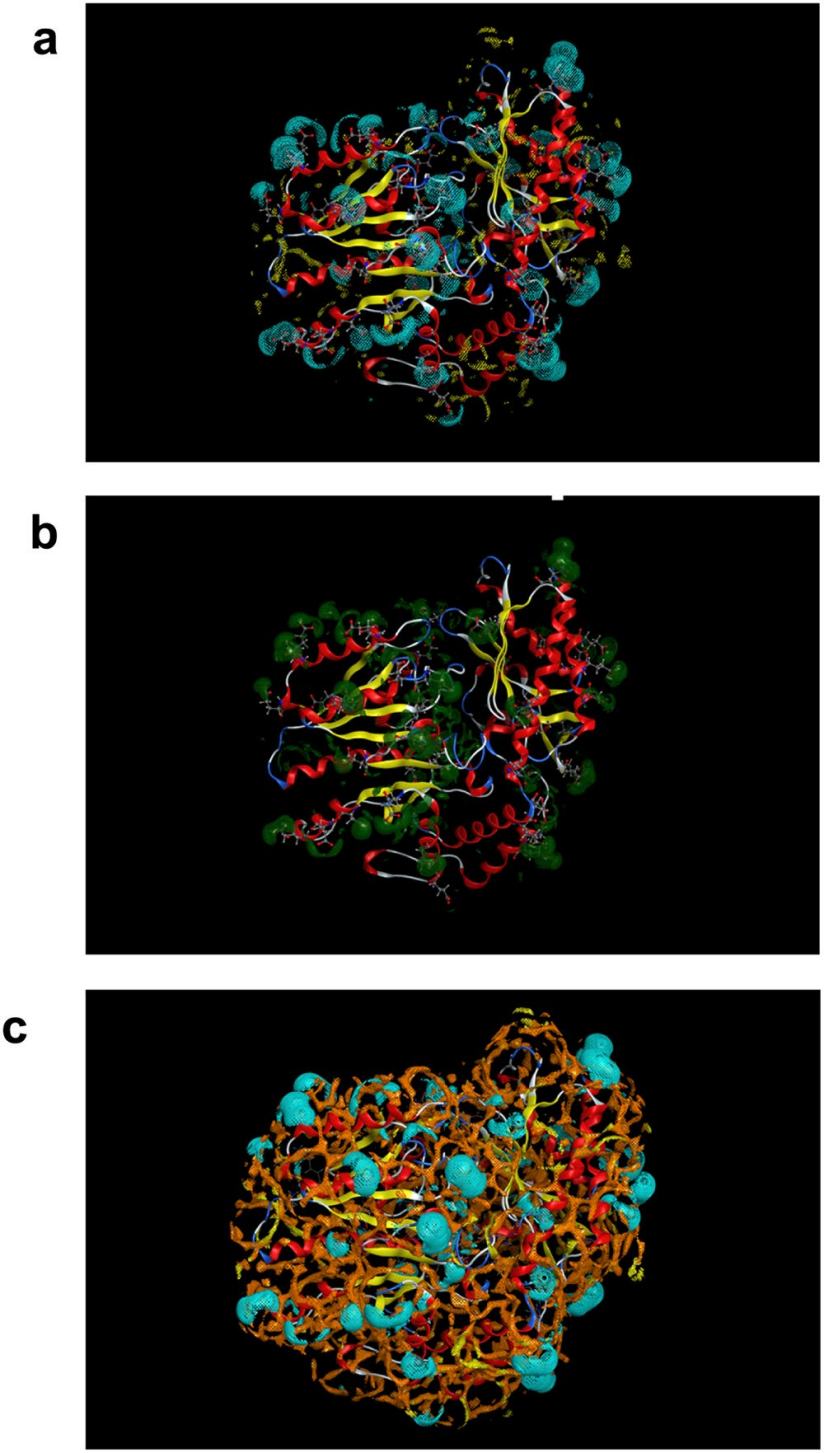
*In silico* solvent analysis of the *Vb*TA T16F surface (**a**) shows the hydration free energy iso-level density (solid green) with a DG cutoff value −5.0 kcal/mol/A3; (**b**) shows the relative sodium and chloride ion iso-levels density relative to an 1 M bulk concentration of NaCl; (**c**) shows both the relative sodium and chloride ion iso-levels density relative to an 1 M bulk concentration of NaCl and the relative hydrophobe (a Lennard-Jones particle, i.e. a probe of the size of a neutral Cl atom) iso-levels density relative to a 50 mM bulk concentration.

The surface area regions of *Vb*TA T16F, with significant hydration free energy contributions (Fig. 6, Panel a, solid green surface), correspond to superficial, negatively charged, glutamic and aspartic acid residues (46 out of 443 total residues). The presence of salt at high concentrations can significantly stabilise *Vb*TA T16F, specifically via extensive interactions with positively charged sodium ions (Fig. 6, Panel b, dotted cyan surface), as can be appreciated from the almost perfect superposition between the hydration free energy and the relative sodium density surfaces. Differently, chloride ions (Fig. 6, Panel b, dotted yellow surface) do not seem to concentrate, to the same extent, around the solvent shell close to *Vb*TA T16F and thus do not play a similar, strong stabilisation role as sodium ions. The Lennard-Jones probe particles (Fig. 6, Panel c, orange surface lines) concentrate all around the *Vb*TA T16F surface, generating a diffused and extended interaction network, but they avoid the high hydration free energy areas (Fig. 6, Panel a, solid green surface) that are preferentially occupied by sodium ions (Fig. 6, Panel b, dotted yellow surface and Panel c, yellow surface lines).

## Conclusion

Our data illustrate how the strategic mutation of a single residue can improve the heterologous expression of an otherwise, unstable/insoluble protein, and represents a strategy that may be adopted for other TA enzymes. This approach was employed to prepare a new transaminase (*Vb*TA) from the highly tolerant marine bacterium *Virgibacillus* sp. 21D. The mutant *Vb*TA T16F was obtained as soluble protein in *E. coli*, and showed remarkable stability towards organic solvents and displayed activity over hydrophobic aromatic substrates (both primary amines and ketones/aldehydes). The crystal structure of *Vb*TA T16F and related computational calculations reveal the crucial structural role of the N-terminal subdomain and the T16F mutation for correct active site architecture and stable dimerisation.

## Materials and Methods

### Marine microorganism, wt *Vb*TA identification, cloning and expression

*Virgibacillus sp*. 21D (EMBL database accession numbers HG799644) was isolated from the water-brine interface of the deep hypersaline anoxic basin Discovery on the Mediterranean Ridge^30^. *Virgibacillus sp*. 21D genome was sequenced and the data were deposited in The Seed database. Genes were annotated using the RAST software42 (Rapid Annotation using Subsytems Technology). *Virgibacillus sp*. 21D was grown in CYSP medium^31^ (casitone 15 g/L, yeast extract 5 g/L, soytone 3 g/L, peptone 2 g/L, MgSO_4_*7H_2_O 15 mg/L, FeCl_3_*6H_2_O 116 mg/L, MnCl_2_*4H_2_O 20 mg/L, NaCl 30 g/L) at 30 °C. After 24 h, cells gown to the stationary phase were harvested and genomic DNA was extracted using the GenElute Bacterial Genomic DNA Kit (Sigma-Aldrich). The *Vb*TA gene was identified by homology, searching *Chromobacterium violaceum*, *Vibrio fluvialis* and *Halomonas elongata* transaminase protein sequences against the *Virgibacillus sp*. 21D genome at default settings of RAST software. All three enzyme sequences matched with a gene annotated as an omega-amino acid-pyruvate aminotransferase. The gene was cloned into pET100-D-TOPO and pET101-D-TOPO plasmid vectors (His tag fused at the N- and C-termini, respectively) (Invitrogen). Expression of the recombinant *Vb*TA protein was performed using BL21(DE3), BL21(DE3)Star, Rosetta(DE3), Codon Plus RIPL *E. coli* strains. *Vb*TA was coexpressed with the pG-KJE8 plasmid that encodes for the chaperonins dnaK, dnaJ, grpE, groES and groEL, according to the manufacturer’s instructions (Takata Clontech). Expression cultures were grown from a single bacterial colony for 24 h at 18, 25, 30, 37 °C, on a rotatory shaker at 200 rpm, in flasks containing LB/TB/ZYM-5052 auto-induction medium^32^ supplemented with 100 µg/mL ampicillin. For LB and TB media, 0.5 mM IPTG was added to induce protein expression at an OD^600*nm*^ of 0.6, inducing for 24 h, unless otherwise stated.

### *Vb*TA T16F cloning, expression and purification

The VbTA gene harboured in a pET100-D-TOPO plasmid was mutated employing the QuikChange Lightning Site-Directed Mutagenesis Kit (Agilent Technologies) using standard protocols. Primers were designed using the QuickChange Primer Design tool (Agilent Technologies).

The best expression conditions were observed using BL21(DE3) cells transformed with the pET100-D-TOPO vector in auto-induction medium^33^. Pellets derived from 300 mL cultures were resuspended in approx. 12 mL (2 mL per g pellet) of wash buffer (50 mM Tris-HCl pH 8.0, 100 mM NaCl, 0.1 mM PLP, 30 mM imidazole) and lysed by sonication, as previously described^17^. The bacterial lysate was clarified by centrifugation at 13,000 × g for 1 h at 4 °C, and filtered using a 0.45 μM filter (Millipore). Using an ÄKTA Start System (GE Healthcare), crude extract was loaded at a flow rate of 1 mL/min into a 1 mL HisTrap HP column packed with NiSO_4_ (0.1 M). The column was washed with wash buffer for 10 column volumes (CVs) and the purified protein was eluted with elution buffer (50 mM Tris-HCl pH 8,0, 100 mM NaCl, 0.1 mM PLP, 300 mM imidazole), following an intermediate wash step with 10 CVs of buffer, prepared by mixing 15% elution buffer with wash buffer, to remove non-specifically bound proteins. Fractions containing the purified enzyme were desalted via overnight dialysis against 50 mM phosphate buffer pH 8, containing 0.1 mM PLP. The purified enzyme was quantified by Epoch Take3 and stored at 4 °C.

### Enzymatic assay

The purified recombinant enzyme was quantified and stored at 4 °C. A kinetic assay derived from Schätzle *et al*.^26^ was used as standard enzymatic assay using pyruvate as the amino acceptor except where stated otherwise. The reactions were carried out at 25 °C employing a reaction mixture containing 1 mL phosphate buffer (50 mM, pH 8), 2.5 mM (*S*)-(–)-1-phenylethylamine, 2.5 mM pyruvate, 0.25% DMSO, 0.1 mM PLP and an appropriate amount of enzyme. These parameters were modified to investigate the behaviour of the enzyme in different reaction conditions. Modifications to the standard assay are indicated when applied. The activity was estimated following the production of acetophenone during the first three minutes of reaction at 245 nm.

### Size exclusion chromatography

The molecular mass of *Vb*TA T16F was estimated by size exclusion chromatography on a Superdex 200 (10/300) GL column with a total bed volume of 24 mL (GE Healthcare), pre-equilibrated with 20 mM phosphate buffer pH 8.0, containing 150 mM NaCl. Elution was carried out at a flow rate of 0.2 mL/min at 4 °C and the column was calibrated using the following MW markers: β-amylase (200 kDa), yeast alcohol dehydrogenase (150 kDa), bovine serum albumin (66 kDa), carbonic anhydrase from bovine erythrocytes (29 kDa), cytochrome c from horse heart (12.4 kDa), Blue Dextran (2000 kDa) was used to calculate the void volume.

### Enzymatic activity

Enzymatic reactions were carried out at 37 °C in potassium phosphate buffer (100 mM, pH 8.0) containing 10 mM amino donor (20 mM if racemic), 10 mM amino acceptor and 0.1 mg/mL of *Vb*TA T16F in a reaction volume of 200 μL. Enzymatic activity under diverse reaction conditions was established employing (*S*)-(–)-1-phenylethylamine as the amino donor and pyruvate as the acceptor. One enzymatic unit was defined as the amount of enzyme that converts 1 μmol of (*S*)-(–)-1-phenylethylamine in 1 min. To study the effect of pH on enzyme activity and stability, a universal buffer was used instead of the phosphate buffer. The effect of co-solvents on *Vb*TA T16F activity and stability was studied by storing the enzyme in the presence of either 10% or 20% (*v/v*) of co-solvent at 4 °C. Before and after incubation the residual activity was determined using the standard spectrophotometric enzymatic assay in presence of the corresponding amount of co-solvents. Each run was performed in triplicate. The kinetic catalytic constants Vmax and Km were measured at pH 8.0 and 25 °C using the two spectrophotometric coupled assays as described above in the presence of 0.25% DMSO, 0.1 mM PLP and 0.1 mg/mL of *Vb*TA T16F. Reactions were carried out with 5 mM pyruvate and varying concentrations (0.5–10 mM) of (*S*)-(–)-1-phenylethylamine, at 5 mM (*S*)-(–)-1-phenylethylamine and various concentrations (0.05–5 mM) of pyruvate. The initial velocity data were fitted to the Michaelis–Menten equation using the SigmaPlot software (Version 11.0).

### HPLC analysis

The final conversion of the different amino acceptors was determined using a Thermo Scientific HPLC instrument equipped with Accucore C18, LC column, Particle size 2.6-micron, diameter 4.6 mm, length 150 mm. Substrates were detected at 210, 245, 280 nm using the following mobile phase A: formic acid (0.1% in water), B: ACN; the gradient elution method adopted was 15% B (10 min), increasing to 80% B (over 8 min), decreasing to 15% B (over 2 min) at 25 °C at a flow rate of 1 mL/min. The depletion of aromatic amines, aldehydes and the formation of acetophenone was evaluated using a calibration curve. Samples were injected after a 1:50 dilution with 0.2% HCl in the quenching step.

### Crystallisation

400 nl crystallisation drops of *Vb*TA T16F solution (5 mg/ml prepared in 20 mM Tris-HCl pH 8.0, containing 2 mM dithiothreitol (DTT) and 50 μM (PLP) were set-up in 96-well round-bottomed sitting drop plates (Greiner), containing 100 μl of 96 Morpheus® screen conditions (Molecular Dimensions). *Vb*TA T16F crystals grew in drops containing 50% *Vb*TA T16F and Morpheus condition D10 (0.12 M Alcohols, 0.1 M Buffer System 3 (Trizma base and bicine, titrated to pH 8.5), 50% *(v/v)* Precipitant Mix 2 (40% *(v/v)* ethylene glycol; 20% *(w/v)* PEG 8000). As all Morpheus® conditions already cryoprotect, crystals were removed directly from the drop, prior to cryocooling in liquid nitrogen.

### Data Collection and Structure Determination

X-ray diffraction data were collected at 2.0 Å resolution on a single *Vb*TA T16F crystal at the automated MASSIF-1 (ID30a1) beamline at the European Synchrotron Radiation Facility (ESRF, Grenoble, France)^34,35^. Data were processed using XDS and assigned to a trigonal (P3_2_21) space group using POINTLESS and scaled using SCALA; both included in the CCP4i suite. The 3D structure of *Vb*TA T16F was solved using Molrep and the structure of a putrescine aminotransferase from *Pseudomonas aeruginosa* (PDB entry 5TI8; 43% sequence identity over 433 aligned residues) as a search model^36^. The structure was manually built using COOT and refined using phenix.refine until satisfactory refinement parameters were achieved (R_work_ = 17.9%; R_free_ = 21.8%). All residues are located in allowed regions of the Ramachandran plot, except for three residues, located in geometrically-restrained regions: S53, located between two β-turns and A283 and the catalytic K284. Data collection parameters and refinement statistics are shown in Supplementary Table 1.

### *In silico* mutagenesis and affinity/stability analysis and calculations

*In silico* mutagenesis simulations and evaluation of protein stability and affinity were carried out using the BioLuminate suite and the OPLS3 force field (Schrödinger, LLC), using the Residue Scanning panel. After residue substitution, the new side chain, surrounding residues and backbone were sampled to the nearest energy minimum and an implicit solvent MM-GBSA method was used to retrieve the solvation energy and the binding affinity of the mutated monomers. The wild type *Vb*TA monomer was generated from the experimental crystallographic structure of *Vb*TA T16F mutant, crystallised as a monomer in the asymmetric unit. The dimer was generated using Chain A and the coordinates for its symmetry-related monomer (180° rotation).

### *In silico* solvent analysis

In order to estimate the effect of the ionic strength on *Vb*TA8 T16F stability, we carried out solvent analyses to characterise the interplay between the solvent (mainly water and salt) and the solute. Calculations were run using the three-dimensional reference interaction site model (3D-RISM) of the Molecular Operating Environment (MOE 2018.01). This application computes a time-averaged distribution of water H and O densities, along with free-energy maps for analysing solvent stability and solvation contributions to binding free-energy.

Calculations were run using the “Solvent analysis” program of the MOE suite and the AMBER10:EHT forcefield. Crystallographic water molecules were removed and VbTA8 was submitted to MOE QuickPrep program before 3D-RISM calculations with 1 M NaCl.

Cut-off values for both the relative sodium and chloride ion densities were set as four-fold denser than bulk and the cut-off value for hydrophobe density was set as two-fold denser than the bulk. In order to favour a graphical comparison between hydration free energy and relative ion and hydrophobe densities, a multiplication factor of 2 was applied to both the relative ion and hydrophobe density grid iso-levels.

### Database

Coordinates and structure factors have been deposited in the Protein Data Bank (www.rcsb.org) under accession number 6FYQ.

## Electronic supplementary material

Supplementary Information
