# Supplementary material for: Strategic single point mutation yields a solvent- and salt-stable transaminase from Virgibacillus sp. in soluble form
Source: Sci Rep. 2018 Nov 6;8:16441. doi: 10.1038/s41598-018-34434-3 (PMC6219536; doi:10.1038/s41598-018-34434-3)
Supplement: Supplementary file 1 — Supplementary Information [file 41598_2018_34434_MOESM1_ESM.docx]

**Supplementary Information**

**Strategic single point mutation yields a solvent- and salt-stable transaminase from *Virgibacillus* sp. in soluble form**

Benedetta Guidi^a^, Matteo Planchestainer^b^, Martina Letizia Contente^b^, Tommaso Laurenzi^d^, Ivano Eberini^d^, Louise J. Gourlay^c^, Diego Romano^a^, Francesca Paradisi^b^, Francesco Molinari^a^

Affiliations:

^a^Department of Food, Environmental and Nutritional Sciences (DeFENS), University of Milan, Milan, Italy

^b^School of Chemistry, University of Nottingham, University Park, Nottingham, NG& 2RD, UK

^c^Department of Biosciences, University of Milan, Milan, Italy

^d^Department of Pharmacological and Biomolecular Sciences (DiSFeB), University of Milan, Milan, Italy


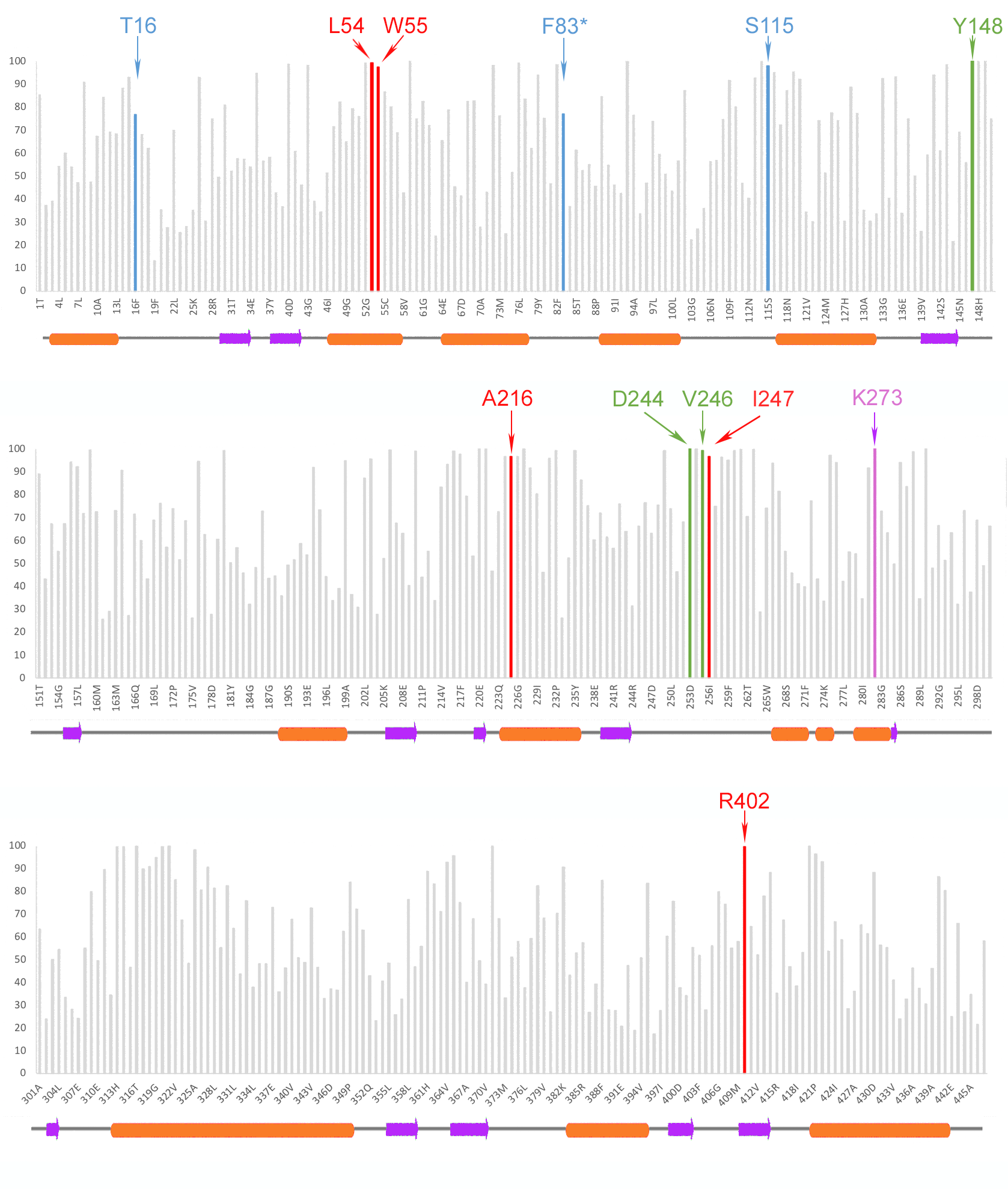


Supplementary Figure 1. ConSurf (<https://www.ncbi.nlm.nih.gov/pmc/articles/PMC2896094/>) results in terms of statistical amino acid frequency for each position.


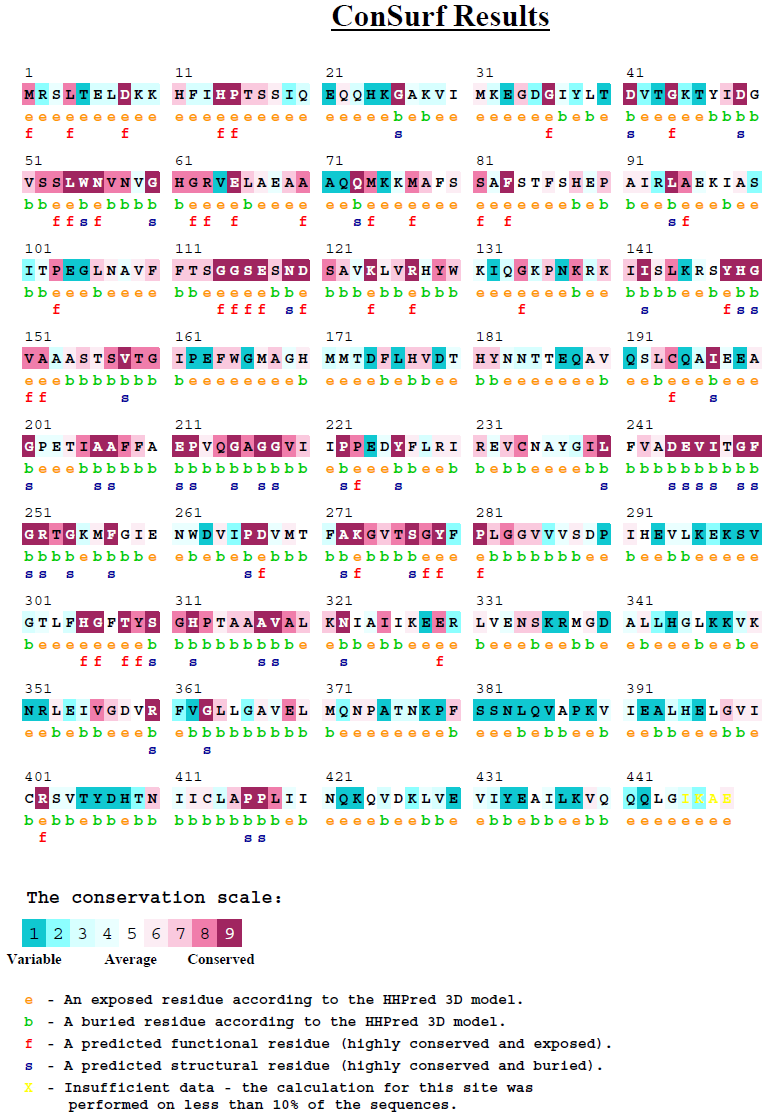


Supplementary Figure 2. Analysis of the conservation of *Vb*TA amino acid residues using ConSurf. The conservation scale calculated by ConSurf (<https://www.ncbi.nlm.nih.gov/pmc/articles/PMC2896094/>) using the full-length *Vb*TA amino acid sequence is shown. Position 16, corresponding to T16, is shown to be significantly conserved.


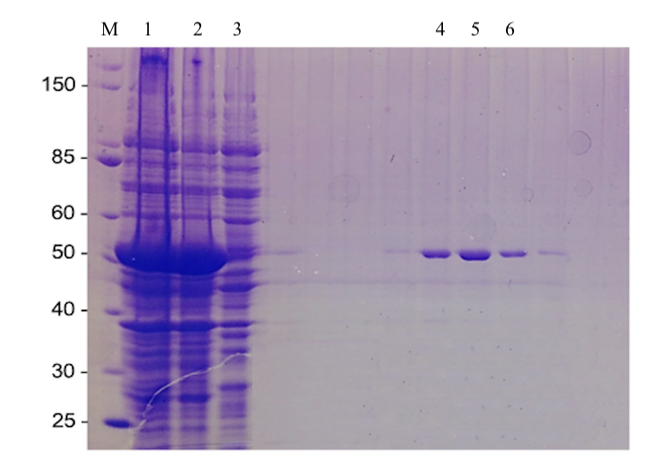


Supplementary Figure 3. SDS-PAGE analysis. Lane M: Protein marker (25-150 kDa); lanes 1 and 2: cell-free extracts; lane 3: affinity column flow-through; lanes 4, 5 and 6: three different purification fractions containing different concentrations of the enzyme expressed in auto induction medium.

**a b**

Supplementary Figure 4. a) *Vb*TA T16F activity and stability at different pHs; b) activity of *Vb*TA T16F at different temperatures, at pH 8.0. Data are reported as relative percentages in comparison with the control in standard conditions and were calculated as an average of triplicate measurements.


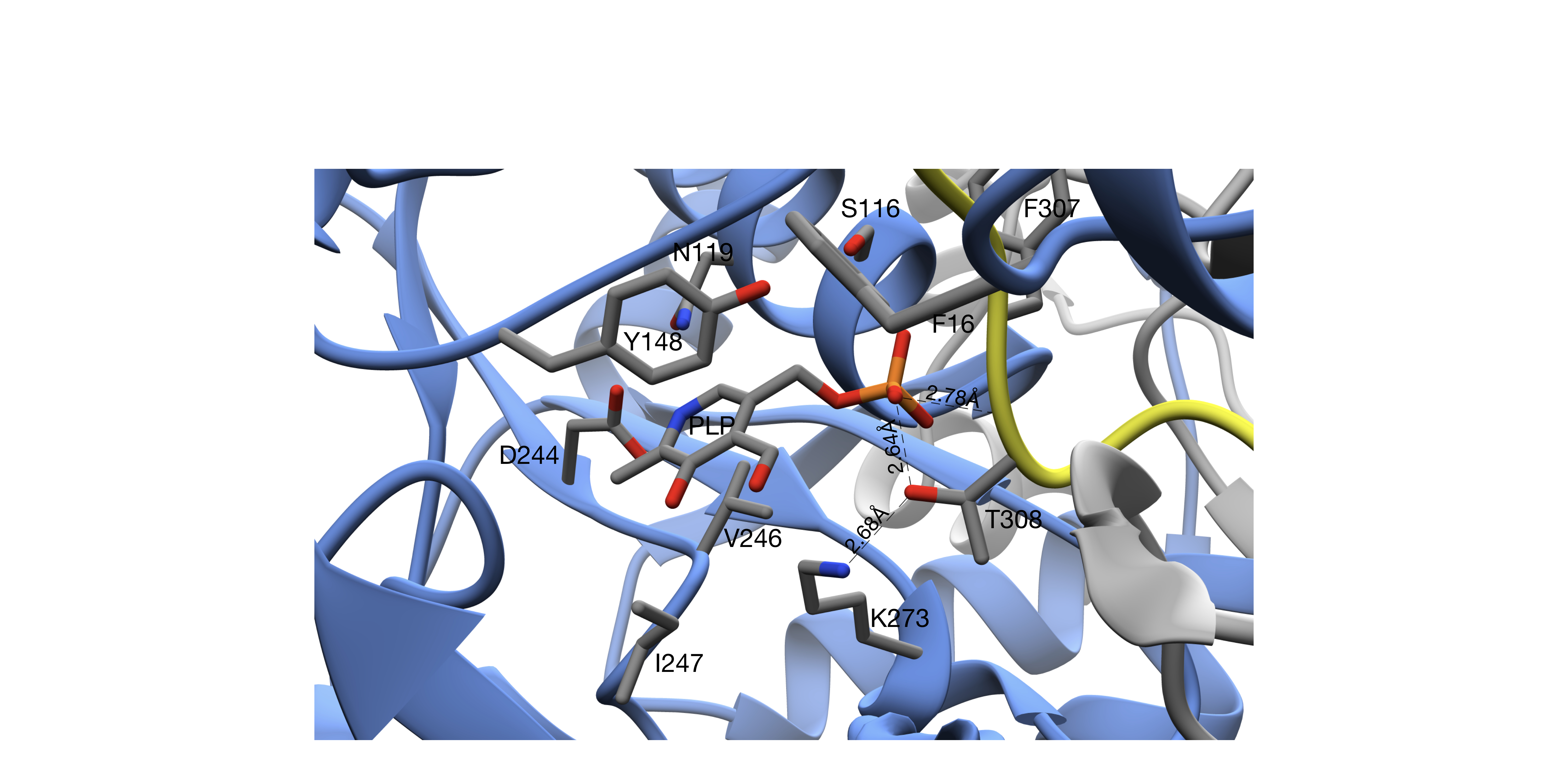


Supplementary Figure 5. Interactions between PLP and *Vb*TA T16F. A detailed view of the active site of chain A of *Vb*TA T16F (blue ribbons) and bound PLP (sticks) is shown. In particular, the stabilising hydrogen bonds formed between T308 (from chain B) and PLP, and with the catalytic lysine residue (K273) are highlighted (dashed lines). Bond lengths are indicated (Å) and the main active site residues that form interactions (< 4 Å) with PLP are shown in sticks and are labelled (for clarity not all residues that form the binding pocket are shown (see Figure 1 for more details). The active site loop of opposing monomer B comprising residues 298-313 is shown in yellow.

S1 **Table. Data collection, refinement and validation parameters for the *Vb*TA T16F crystal**. R_merge_ = ∑⏐I-*(I)*/ ∑ I x 100, where I is the intensity of a reflection and *(I)* is the average intensity; Values in parentheses represent data belonging to highest resolution shells (2.0-2.11 Å). Data were collected on a single crystal.

|  | ***Vb*TA** **T16F** |
| --- | --- |
| **Data collection** |  |
| Space group | P3_2_21 |
| Cell dimensions |  |
| *a*, *b*, *c* (Å) | 100.9 100.9 97.4 |
|  α, β, γ (°) | 90, 90, 120 |
| Resolution (Å) | 40-2.0 |
| *R*_merge_ | 0.046 (0.45) |
| *I* / δ | 17.3 (3.2) |
| No. unique reflections | 38942 (5629) |
| Completeness (%)  Redundancy | 99.6 (99.9)  3.8 (3.9) |
|  |  |
| **Refinement** |  |
| Resolution (Å) | 40-2.0 |
| *R*_work_ / *R*_free_ (%) | 17.9/21.9 |
| *No. atoms* |  |
| Protein | 3417 |
| PLP | 24 |
| Water | 74 |
| Ethylene glycol | 30 |
| Chloride ion | 1 |
| *B-*factors (Å^2^) |  |
| Protein | 50.1 |
| PLP | 38.6 |
| Water | 40.7 |
| Ethylene glycol | 70.3 |
| Chloride ion | 83.9 |
| *R.m.s. deviations* |  |
| Bond lengths (Å) | 0.011 |
| Bond angles (°) | 1.088 |
| *Ramachandran Plot (%)*  Favored Regions  Allowed Regions | 95.5  99.3 |
